# Latent groups from multidimensional factors and effects on suicidal ideation in older adults

**DOI:** 10.1192/bjo.2024.60

**Published:** 2024-05-07

**Authors:** Hyun Lee, Eunjin Lee

**Affiliations:** Department of Social Welfare, Mokwon University, Daejeon, South Korea; The Center for Social Welfare Research, Yonsei University, Seoul, South Korea

**Keywords:** Multidimensional risk, older adult, suicidal ideation, latent profiles analysis

## Abstract

**Background:**

Suicide is a serious social problem among older adults. However, little is known about how multidimensional factors affect suicide of older adults.

**Aims:**

We classify the multidimensional suicidal risk types of older adults based on their characteristics and identify differences in suicidal ideation.

**Method:**

Data were obtained via a nationwide online survey targeting 612 older adults over the age of 55 years. A latent profile analysis identified three profiles, one of which represented the optimal situation for these adults.

**Results:**

We identified three distinct multidimensional suicidal risk types in older adults: high-risk predicament (24.5%), moderate-risk predicament (57.7%) and abundant internal/external resources (17.8%). In particular, depression, a major risk factor for suicide, was found together with self-neglect in each group. Multiple regression analysis showed that older adults in the moderate-risk predicament and high-risk predicament groups were more likely to have suicidal ideation than those in the abundant internal/external resources group.

**Conclusions:**

Our findings suggest that co-occurrence of depression and self-neglect represents a suicide risk pattern in high-risk older individuals. Therefore, local communities need to urgently screen and provide interventions for such older adults and strengthen their capacity for multidimensional aspects of life to prevent suicide in the long term.

Suicide is a serious social problem. In 2020, Korea had the world's highest suicide rate, at 23.5 deaths per 100 000, compared with the OECD average of 10.9 deaths per 100 000^1^ Moreover, the average suicide rate among Koreans in their 50s or older is very high, exceeding 30 deaths per 100 000.^1^ There is thus an urgent need to identify older adults vulnerable to suicide and determine their characteristics. Studies have been conducted to identify the characteristics of individuals or groups at higher risk of suicide based on risk factors associated with elderly suicide. A systemic review on suicidal behaviour in older age found that risk factors most closely associated with self-harm attempts in elderly individuals included depressive disorders, methods of self-harm, and use of psychotropic medications. In particular, male sex, violent methods of self-harm, any psychiatric disorder, poor medical condition, stressors/bereavement and living alone appeared to be significant predictors of suicide.^2^ In addition, other studies have reported that individual social factors such as limited social connectedness,^3^ low social trust^4^ and low social participation^5^ have a great impact on suicide of elderly people. These studies contribute to identifying risk and protective factors influencing elderly suicide based on various research outcomes. However, they were limited by not being able to simultaneously verify each factor. Consequently, it is challenging to understand which groups are most vulnerable to suicide based on variations in these factors.

Recent studies have used latent class analysis (LCA) or latent profile analysis (LPA) to classify individuals into groups vulnerable to suicide. As an example, in the study by Podlogar et al., LCA was applied to classify suicide risky groups among psychiatric outpatients based on depression and anxiety indicators.^6^ Subgroups with high levels of depression and physical anxiety were found to have a high risk of suicide. Also, another study used LPA to classify older adults living alone in Korea into three types based on psychosocial risk factors.^7^ The study found a positive correlation between depression and suicidal ideation and a negative correlation between low social support and high depression and loneliness. However, few studies have focused on older adults. In addition, most studies of suicide have focused on only a small number of mental health or psychological indicators;^8^ hence, they have failed to consider social-relational and community factors in an integrated manner.

Older adults face various changes and difficulties, and complex long-term factors are involved in the causes of suicide in this population.^9^ Thus, the suicide problem among older adults cannot be addressed by intervening with respect to only one factor.^10^ According to Leenaars’ multidimensional model, suicide is a result of a multidimensional process involving individuals’ psychosocial internal and external factors.^9^ This is similar to the ‘predicament model’ of suicide, which divides the stressor factors of suicide into internal predicaments related to mental health and intrapsychic factors and external predicaments explained by the external environmental and situational factors.^11^ Taking a multidimensional approach, this study considered the external and internal indicators generated by prior suicide-related studies to identify older adult groups vulnerable to suicidal ideation.

Numerous studies have identified internal factors related to intrapsychic and mental health that influence suicide.^12^ Depression is reported to be the strongest and most direct intrapsychic factor in suicide.^13^ Moreover, depression is highly associated with and accompanied by other intrapsychic factors such as anxiety, other mental disorders and psychological distress, which all affect suicide.^14^ In addition, the absence or impairment of goals, and the presence of hopelessness and negative cognitive distortions about reality and the future^15^ are intrapsychic risk factors leading to suicidal ideation. However, in addition to focusing on these risk factors, a new approach has been used to investigate effective protective factors that can reduce the potential risk of suicidal ideation and behaviour. Meaning in life,^16^ hope^17^ and self-compassion, which enable one to adapt to difficult situations and overcome suicidal crises as part of an inner resilience,^18^ are among the protective factors that have been confirmed to have an impact. Self-compassion refers to the intrinsic psychological capacity of mindful awareness, where individuals do not avoid or evade the pain and difficulties they experience.^19^ It involves recognising these challenges not as issues exclusive to oneself but as problems that anyone can encounter, alleviating the suffering through a mindset that acknowledges the universality of such experiences.^20^ This study considered depression (which is the strongest risk factor for suicide), motivation and self-compassion, which are internal protective factors, to be intrapsychic indicators affecting suicide.

The external risk factors influencing suicide are closely related to social connectedness.^8^ Social connectedness is a concept that includes not only interpersonal relationships with family and friends but also a sense of belonging to the community, such as social participation and social trust. It directly impacts elderly suicide.^21^ In addition, unmet expectations and frustrated ambitions with respect to employment, health and financial status are among the external stressors influencing suicide.^15^ Self-directed aggression and escape behaviours or attitudes occur when needs for relational, environmental and physical factors are not fulfilled; this can lead to suicide.^15^ Self-neglect refers to the inability to perform essential self-care tasks, indicating a state where individuals cannot meet their basic needs.^22^ This is manifested through behaviours that threaten the health and safety of older people. It involves the inability to secure essential physical living conditions and personal hygiene conditions, manage one's finances, and obtain goods and services for maintaining physical health, emotional stability and general safety, leading to social isolation.^22,23^ This study considered health status, social support, social participation, social trust and self-neglect to be external indicators related to the social/environmental factors affecting suicide.

Intrinsic and extrinsic factors that affect suicidal ideation are reported to have different effects according to individual sociodemographic characteristics. In general, suicidal ideation is higher with older age^24^ and occurs more in elderly women than in men.^24,25^ Considering marital status, being non-married/non-cohabiting had a significant effect on suicidal ideation.^26^ Absence of a spouse in old age owing to bereavement or divorce is linked to living alone and can lead to a decrease in interpersonal networks and support systems, which can negatively affect suicidal ideation.^27^ Low socioeconomic status (SES) is an established risk factor for suicidal behaviors.^28^ Education and working status are variables closely related to SES,^29^ and low levels of educational attainment and unemployment have a significant effect on suicidal ideation in older adults.^25^ Based on previous studies, the present study attempted to examine differences in the effects of gender, age, employment status, education level, marital status and SES on subgroups of suicidal ideation classified using LPA.

To review, suicidal ideation among older adults is the result of multidimensional and complex action of external/internal factors, and their patterns differ depending on the individual. We classified types of potential suicide crises using LPA based on the characteristics of the external/internal factors that affect suicidal ideation. LPA is an analytical approach that categorises groups based on the similarities of response patterns among individuals who are heterogeneously latent in the collected data; it is applied when the indicators used for analysis consist of continuous variables.^30^ Therefore, it is an appropriate method for classifying suicide risk groups based on the characteristics of individual responses. After applying LPA, we examined the characteristics of the subgroups that were typified and checked the differences in the impacts of each type on suicidal ideation. Overall, we explored which older adult groups with which characteristics were most vulnerable to suicidal ideation. The aim was to propose ways of reducing suicidal ideation in older adults by considering the characteristics of each type of suicidal crisis.

## Method

### Participants and procedure

We conducted a nationwide online survey targeting 612 older adults over the age of 55 years. The nature of online surveys means that they are likely to receive more responses from relatively young age groups, so quota sampling was performed by age group (256 people 55 to 64 years old, 356 people 65 years old or older) based on the population ratio. We set the minimum age to 55 years because this is the retirement age set by most Korean companies; therefore, this is when retirement and lifestyle changes usually occur (Korea's average retirement age was recently calculated to be 49.3 years).^31^ The survey was conducted for about 3 weeks from 13 October to 3 November 2021. After respondents expressed their intention to participate voluntarily, they completed the survey using a smartphone or PC. The research goals and contents were explained in advance of the survey, and only individuals who clicked on the consent to participate in the study were allowed to respond. In addition, respondents were informed that they could stop participating in the survey at any time if they wished. As the survey response data were encrypted and converted into personally identifiable data, personal information was thoroughly protected. The authors assert that all procedures contributing to this work comply with the ethical standards of the relevant national and institutional committees on human experimentation and with the Helsinki Declaration of 1975, as revised in 2008. All procedures involving human subjects/patients were approved by Yonsei University Institutional Review Board (7001988-202110-HR-1380-02).

### Measures

The Suicide Ideation Scale was used to measure suicidal ideation.^32^ It consists of five questions on suicidal thoughts and suicide attempts. Regarding its reliability, the Cronbach's alpha was 0.84. To measure self-neglect among the elderly, the Self-Neglect Scale for older adults was used;^33^ the Cronbach's alpha was 0.88. To measure self-compassion, the Korean version of the Self-Compassion Scale^34^ was used; its Cronbach's alpha was 0.72. For motivation, the items to evaluate intrinsic motivation were used;^35^ the Cronbach's alpha was 0.94. For depression, a five-item version of the Geriatric Depression Scale was used;^36^ its reliability was 0.79. Social trust was measured as a single item using the question: ‘To what extent do you think our society is reliable?’ Answers ranged from ‘not at all’ (1) to ‘very reliable’ (5).^37^ Social support was measured as the number of people (e.g. family members, relatives, friends, neighbours and colleagues) who could provide help. If no one could provide help, this was marked as 0. Regarding health, the respondents were asked to choose from ‘very bad’ (1) to ‘very healthy’ (5) in answer to the question ‘How is your health in general?’ A single question was used for social participation. The respondents were asked, ‘Have you ever participated in one or more social groups (social groups, religious groups, leisure activity groups, etc.) during the past year?’ The answers ranged from ‘not participated at all’ (1) to ‘very actively participated’ (4).

### Data analysis

We conducted the analysis in three steps using LPA. In the first step, we identified the optimal number of potential profiles based on the relevant index. Various indices, including log-likelihood, Akaike's information criterion (AIC), Bayesian information criterion (BIC), sample-size-adjusted BIC (SABIC), and the entropy and Vuong-Lo-Mendell-Rubin likelihood ratio test (LMR-LRT), were used to select the number of profiles. Lower values of log-likelihood, AIC, BIC, and SABIC and higher values of entropy were interpreted as being more suitable. Significance in LMR-LRT indicates that the *k*-profile is a more compact solution than the *k*−1-profile.^38^ The following indicators were used for profile classification. Internal aspects included indicators of depression, motivation and self-compassion, and external aspects encompassed indicators of health, social support, social participation, social trust and self-neglect. In the second step, chi-squared test and analysis of variance (ANOVA) were used to explore the sociodemographic variables of the survey respondents and predictors of suicidal ideation. In the third step, after controlling for sociodemographic variables, we performed multiple regression analysis to verify the influence of suicidal ideation on groups derived from the profile analysis. The statistical procedures were performed using R software and the tidyLPA package^39^ for LPA.

## Results

### Latent profile analysis

Table 1 summarises the classification of multidimensional suicidal risk types among older adults. From classes 2 to 5, the log-likelihood, AIC and SABIC values continued to decrease, and the LMR-LRT values were significant in all classes. However, class 3 showed the lowest BIC value and a greater entropy value. When these results were considered comprehensively, class 3 was identified as representing the optimal situation.

**Table 1:** Goodness-of-fit statistics for classifications

Class	Log-likelihood	AIC	BIC	SABIC	Entropy	LMR-LRT	*P*-value
1	−4915.91	9863.83	9934.49	9883.70	1.00		
2	−4741.52	9533.04	9643.46	9564.09	0.83	348.78	0.01
3	−4659.24	9386.47	9536.64	9428.70	0.75	164.57	0.01
4	−4635.78	9357.56	9547.48	9410.96	0.71	46.92	0.01
5	−4621.47	9346.95	9576.62	9411.53	0.72	28.61	0.01

AIC, Akaike information criterion; BIC, Bayesian information criterion; SABIC, sample-size-adjusted BIC; LMR-LRT, Vuong-Lo-Mendell-Rubin likelihood ratio test.

**Fig. 1:**
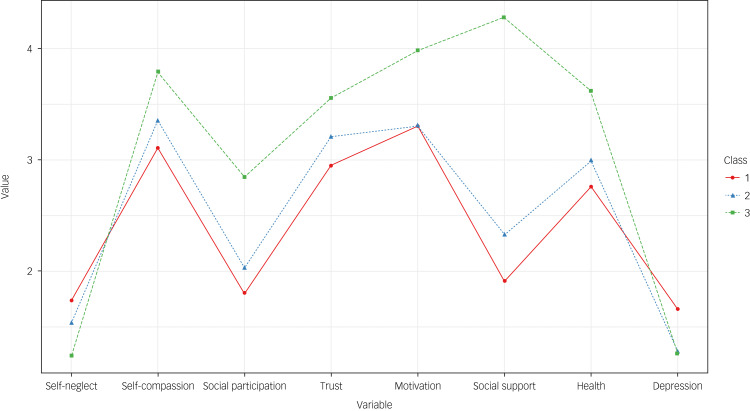
Multidimensional suicidal risk groups in the three-class latent profile model. Class 1, high-risk predicament; class 2, moderate-risk predicament; class 3, abundant internal/external resources.

Figure 1 shows the characteristics of the multidimensional suicidal risk types of older adults. The group with high depression and self-neglect and low motivation and social participation was named the ‘high-risk predicament’ group (24.5% of the sample). Class 2, with moderate psychosocial risks scores, was classified as the ‘moderate-risk predicament’ group (57.7% of the sample). Class 3, with high self-compassion, motivation, social participation and trust and low depression and self-neglect was classified as the ‘abundant internal/external resources’ group (17.8% of the sample).

### Predictors of class membership

Table 2 presents the differences in sociodemographic and suicidal ideation variables among the three multidimensional suicidal risk types. Chi-squared test and one-way ANOVA were performed according to the nature of the variables. Significant differences between classes were observed with respect to education, marriage, SES and suicidal ideation. The ‘abundant internal/external resources’ profile had a higher education level compared with the other profiles. Married/cohabiting individuals accounted for a rather low proportion of the ‘high-risk predicament’ profile, whereas their proportion was relatively high in the other profiles. The highest SES and the lowest suicidal ideation were found for the ‘abundant internal/external resources’ profile (mean = 5.8, s.d. = 1.5; mean = 1.4, s.d. = 0.5, respectively). No class differences were observed in terms of gender, age group or work.

**Table 2:** Differences in sociodemographic and suicidal ideation by profile

	High-risk predicament,^a^ *N* = 150 (24.5%)	Moderate-risk predicament,^a^ *N* = 353 (57.7%)	Abundant internal/external resources,^a^ *N* = 109 (17.8%)	Significance test (*post hoc*)^b^
Gender				χ^2^(2) = 0.69
Male	57 (38.0%)	141 (39.9%)	47 (43.1%)	
Female	93 (62.0%)	212 (60.1%)	62 (56.9%)	
Age group				χ^2^(2) = 3.13
Early elderly	72 (48.0%)	140 (39.7%)	44 (40.4%)	
Late elderly	78 (52.0%)	213 (60.3%)	65 (59.6%)	
Work				χ^2^(2) = 4.26
Worker	78 (52.0%)	218 (61.8%)	66 (60.6%)	
Non-worker	72 (48.0%)	135 (38.2%)	43 (39.4%)	
Education				χ^2^(2) = 28.44***
Less than high school	81 (54.0%)	149 (42.2%)	23 (21.1%)	
Higher than high school	69 (46.0%)	204 (57.8%)	86 (78.9%)	
Marriage				χ^2^(2) = 8.52*
Married/cohabiting	116 (77.3%)	302 (85.6%)	98 (89.9%)	
Others (separation, divorce, bereavement, etc.)	34 (22.7%)	51 (14.4%)	11 (10.1%)	
Socioeconomic status	4.5 ± 1.8	5.0 ± 1.6	5.8 ± 1.5	F(2, 609) = 18.79***
Suicidal ideation	2.3 ± 0.8	1.7 ± 0.7	1.4 ± 0.5	F(2, 609) = 53.71***

a. Values are frequency (percentage) or mean (standard deviation).

b. *P*-values come from chi-squared test or analysis of variance.

*P < 0.05, **P < 0.01, ***P < 0.001.

### Multiple regression for suicidal ideation

Multiple regression analysis was performed to verify the effects of the multidimensional suicidal risk types on suicidal ideation among older adults (Table 3). The ‘abundant internal/external resources’ profile was used as the reference group for comparison with other groups. The other groups (moderate-risk predicament and high-risk predicament) had significantly higher rates of suicidal ideation than the reference group. In particular, the older adults with the ‘high-risk predicament’ profile showed the greatest effect size for suicidal ideation (β = 1.13, *P* < 0.001).

**Table 3:** Multiple regression for suicidal ideation

	Suicidal ideation			
Predictors	Estimates	Beta^a^	CI	*P*-value
Gender				
Male	Reference	–	–	–
Female	−0.00	−0.00	−0.12 to 0.12	0.975
Age group				
Early elderly	Reference	–	–	–
Late elderly	0.07	0.09	−0.05 to 0.19	0.229
Work				
Worker	Reference	–	–	–
Non-worker	−0.03	−0.03	−0.15 to 0.09	0.661
Education				
Less than high school	Reference	–	–	–
Higher than high school	0.01	0.02	−0.11 to 0.14	0.821
Marriage				
Married/cohabiting	Reference	–	–	–
Others (separation, divorce, bereavement, etc.)	−0.01	−0.01	−0.17 to 0.16	0.924
Socioeconomic status	−0.03	−0.07	−0.07 to 0.01	0.096
Cluster				
Abundant internal/external resources	Reference	–	–	–
Moderate-risk predicament	0.34	0.43	0.18–0.50	<0.001
High-risk predicament	0.88	1.13	0.69–1.06	<0.001

a. Standardised coefficient.

## Discussion

In this study, first, the participants were classified into ‘high-risk predicament’, ‘moderate-risk predicament’ and ‘abundant internal/external resources’ groups according to the results of LPA based on multidimensional factors affecting suicidal ideation. Unlike the other two types, the ‘abundant internal/external resources’ type was associated with high self-compassion, social participation, health, motivation, social support and social trust but markedly low depression and self-neglect. For the ‘high-risk predicament’ profile, levels of depression and self-neglect were high, but those of self-compassion and health were low. For the ‘moderate-risk predicament’ profile, all variables showed moderate levels of psychosocial risk.

Depression, one of the major characteristics, tended to appear along with self-neglect in both the ‘high-risk predicament’ and ‘abundant internal/external resources’ groups. However, for the ‘high-risk predicament’ profile, depression and self-neglect levels were both found to be very high, whereas the in ‘abundant internal/external resources’ profile, depression and self-neglect levels were low. It was thus confirmed that depression occurs with self-neglect. Suicidal ideation was found to be highest when depression and self-neglect had a high combined effect. Previous studies have verified that depression has a positive correlation with self-neglect.^40^ Thus, the combined effect of depression and self-neglect observed in the ‘high-risk predicament’ group suggests that depression influenced self-neglect. Future research should verify the combined effect of depression and self-neglect on suicidal ideation.

Second, comparing the sociodemographic characteristics of subgroups classified through LPA revealed statistically significant differences in education, marriage and SES. These results are consistent with those of previous studies mentioned above.^20–24^ Therefore, to understand suicidal ideation in vulnerable groups, it is necessary to focus on these characteristics, with close attention to the risk of suicide. In the ‘high-risk predicament’ group – which showed very low levels of social support, social participation and social trust – the ratio of people without a spouse was much higher than in the other two groups. This result is in line with the findings of previous studies on the social relationships of older adults who committed suicide, which found that they were more likely to live alone and have fewer acquaintances and friends, as well as being less likely to participate in social activities and having low levels of social trust.^41^ Moreover, a study that attempted to typify the psychosocial crisis in older adults using LPA found that low social support was accompanied by high loneliness and depression.^7^ When planning suicide prevention policies, it is necessary to ensure that they enhance social support and social trust and increase social participation, taking into account the suicide risk of older adults without a spouse or with weak social networks and socioeconomic resources.^42^

In Korea, a specialised service in the Individualised Support Service for older adults is provided to prevent lonely death and suicide among vulnerable elderly people with high levels of social isolation and depression. The recipients of this service are divided into two groups: depressed older adults who suffer from mental health problems and are at high risk of suicide; and reclusive older adults who are cut off from family and neighbours, and disconnected from public and private support systems. Specialised services not only provide mental health treatment for the group at high risk of depression and suicide but also identify older adults who are disconnected from society and promote connection through individual case management strategies such as counselling, group activities, medical treatment, connection to community resources, and so on. However, as voluntary application for this service is likely to be low, social efforts are needed to identify and find people in need. Based on the results of this study, among low-income elderly people living alone, those who do not use welfare centres or senior citizen centre or have no local community activities, could be screened first in efforts to identify high-risk older adults with limited social networks.

Third, this study compared the influences on suicide between the groups through multiple regression analysis. When the ‘abundant internal/external resources’ group was used as a reference, the ‘high-risk predicament’ profile showed a strong effect on suicidal ideation based on the standardised coefficient. This result could be used as a basis to determine which group needs intervention first. Previous studies have found that suicidal ideation in older adults is highly likely to lead to suicide attempts and completed suicide.^38,43^ Therefore, the ‘high-risk predicament’ group, which showed the highest level of suicidal ideation, can be viewed as a high-risk group with a high probability of suicide implementation. Therefore, local communities need to urgently screen and provide interventions for older adults who show the characteristics of the ‘high-risk predicament’ group.

The ‘high-risk predicament’ group also had the highest levels of depression and self-neglect. Therefore, using depression and self-neglect assessments may be an effective way to screen older adults who can be classified as having a ‘high-risk predicament’ profile. Furthermore, as mentioned earlier, owing to the higher rates of suicide attempts and completions among older adults with high suicidal ideation, early intervention is essential for at-risk groups. Klonsky and May's three-step theory^44^ suggests that psychological pain and hopelessness contribute to suicidal ideation, whereas lack of connectedness influences its escalation, potentially leading to suicide attempts. Therefore, proactive early intervention is vital after identifying groups with high levels of suicidal ideation, focusing on reducing depression influenced by psychological pain and hopelessness, as well as mitigating self-isolating and neglectful behaviours that affect connectedness. To address this specifically, the significance of community-based education programmes is emphasised, aiming to raise awareness of potential environmental hazards and promote self-care, thereby preventing older adults from experiencing depression and engaging in self-harm.^45^

Conversely, the ‘abundant internal/external resources’ group, with the lowest risk of suicidal ideation, showed high self-compassion and motivation (internal indicators). This group also displayed high levels of health, social participation, social support and social trust (external indicators). Therefore, for older adults in the ‘abundant internal/external resources’ group, interventions could focus on managing and strengthening these protective factors for suicide prevention.

This study had several limitations. First, it used data collected through an online survey targeting older adults, and the questions were kept short to increase the response rate and enable intuitive responses. In future research, additional analysis using standardised scales is needed to more precisely confirm the effects of the variables considered in this study.

Second, the health-related variables used in this study were measured subjectively and did not reflect the objective health status of the participants. More research is needed to understand the effects of objective health conditions such as frailty and functioning disability, chronic diseases and cognitive disorders on suicidal ideation. Recently, studies have used biomarkers to verify causality in suicide risk or suicidal behavior.^46^ More precise verification will be possible if objective health-related indices and variables are used.

Third, LPA was conducted, and the effects of typified latent profiles on suicidal ideation were examined in a cross-sectional study. The study did not fully investigate the longitudinal causal relationships between changes in the latent profiles over time and suicidal ideation. It is important to understand these longitudinal relationships using analyses such as latent transition analysis and latent class growth analysis. More research is needed using longitudinal data to determine whether transitions occur over time in different types of biopsychosocial crisis. Research that identifies the longitudinal effects of these types on suicidal ideation could better reflect the characteristics of suicide among older adults, which evolve over a long period, from the stages of suicidal ideation and suicide planning to final implementation.

## Data Availability

The data-sets analysed during the current study are not publicly available. Any individual may apply for data access by contacting the corresponding author.
